# Phonetic accommodation depends on processing conditions: evidence for task-dependent frequency effects

**DOI:** 10.3389/fpsyg.2026.1885387

**Published:** 2026-09-04

**Authors:** Mitko Sabev, Ivan Yuen, Bistra Andreeva, Bernd Möbius

**Affiliations:** Department of Language Science and Technology, Saarland University, Saarbrücken, Germany

**Keywords:** gender, German *-ig* suffix, input–output coupling, lexical frequency, phonetic accommodation, processing conditions, task effects

## Abstract

This study investigates how lexical frequency, behavioural task, and gender shape phonetic accommodation, focusing on speakers' adoption of an alternative pronunciation variant of the German adjectival suffix *-ig*. Partially overlapping groups of 30–31 native speakers of German completed two experimental tasks: a shadowing task involving immediate repetition of the same target adjective, and an interaction task based on question–answer exchanges in which the perceived and produced *-ig* adjectives were different lexical items but belonged to the same lexical-frequency category. Lexical frequency was manipulated by selecting high- and low-frequency stimuli, and accommodation was operationalised as a categorical shift from each speaker's dominant baseline pronunciation towards the alternative variant presented in the stimulus. Accommodation behaviour was analysed using a generalised linear mixed-effects model. The results show that accommodation increased with exposure, was stronger in shadowing than in interaction, and was greater overall for women than for men. Accommodation also differed depending on the direction of change, whereas stimulus voice gender did not play a role. Crucially, lexical frequency affected accommodation only in the shadowing task: low-frequency items elicited greater accommodation than high-frequency items, while no comparable frequency effect emerged in interaction. This task-dependent pattern suggests that lexical-frequency effects are strongest when perceived and produced forms are temporally adjacent and lexically identical, as in immediate repetition of the same word. When accommodation requires generalisation across different words sharing only the same suffix, as in the interaction task, frequency effects may be attenuated or fail to emerge. These findings support a hybrid account in which accommodation reflects the interaction of the link between perceived and produced forms, task-specific processing demands, lexical frequency, and speaker-related factors.

## 1 Introduction

Phonetic accommodation refers to a well-documented tendency for speakers to adapt aspects of their speech to that of their interlocutors. This adaptation can occur across a wide range of acoustic-phonetic and phonological dimensions, including speech rate, vowel formant frequencies, voice onset time, pronunciation variant selection, and prosodic features (Pardo et al., 2022). While the existence of accommodation is robustly established, the mechanisms driving it remain a matter of ongoing debate. Some researchers view convergence and divergence as socially motivated, reflecting speakers' desire to signal affiliation or distance. Others attribute convergence to priming or alignment processes, often characterised as automatic, arising from relatively direct links between speech perception and speech production. Yet others have argued for hybrid accounts in which social and automatic mechanisms jointly shape accommodation behaviour.

Despite substantial theoretical progress, an important dimension is still incompletely understood: the role of lexical frequency in phonetic accommodation. Lexical frequency may influence accommodation through representational mechanisms such as episodic-memory dynamics and frequency-based entrenchment. The central question investigated in this study is whether and under what processing conditions lexical frequency modulates speakers' adoption of an alternative pronunciation variant. We address this question by examining accommodation in the production of the German adjectival suffix *-ig*, which exhibits well-known pronunciation variation between a fricative ([ɪç]) and a plosive ([ɪk]) variant. By combining an experimental manipulation of lexical frequency with two distinct task types—shadowing and interaction—the study examines whether frequency effects are strongest when a recently perceived form can influence subsequent production of the same lexical item with minimal intervening processing. The prediction is that a recently perceived variant should have greater relative weight for low-frequency words, whose representations are less densely supported by previous experience, than for high-frequency words. We argue that lexical frequency does not exert a general influence on accommodation, but operates conditionally, emerging most clearly under task conditions in which input and output are closely matched.

## 2 Background

### 2.1 Phonetic accommodation: social, automatic, and hybrid accounts

Phonetic accommodation has been approached from several theoretical perspectives, which differ in the extent to which convergence and divergence are attributed to socially motivated behaviour or to relatively automatic links between speech perception and speech production (for reviews, see Pardo et al., 2022; Pellegrino et al., 2024). Socially oriented accounts treat accommodation as a meaningful aspect of interaction: convergence may signal affiliation, solidarity, or group alignment, whereas divergence may signal social distance, distinct identity, or resistance. In contrast, automatic accounts attribute convergence to priming or alignment mechanisms, whereby recently perceived linguistic forms activate representations or routines that can influence subsequent production, even without explicit intention to imitate.

Neither perspective on its own provides a complete account of the full range of observed accommodation patterns. Convergence is highly variable across speakers, phonetic features, tasks, and contexts: speakers may converge on some phonetic dimensions while diverging on others, and convergence in one domain does not necessarily predict convergence in another. Such variability suggests that accommodation is shaped by multiple interacting factors, including phonetic distance between interlocutors, lexical frequency, cognitive load, personality traits, task demands, and social evaluation (Pardo et al., 2022; Pellegrino et al., 2024).

The present study adopts a hybrid perspective, in which accommodation is understood as the outcome of interactions between recent-input priming, representational constraints, task-specific processing demands, and social or speaker-related factors (Babel, 2010, 2012; Coles-Harris, 2017; Gessinger et al., 2021; Krauss and Pardo, 2004; Pardo, 2012). Within this broader view, exemplar-based and episodic-memory mechanisms are especially relevant to the present focus on lexical frequency. Under such approaches, spoken words are represented not only by abstract lexical entries, but also by detailed traces of previous encounters with those words (Goldinger, 1998; Pierrehumbert, 2001). Frequency is crucial in such models because high-frequency words are associated with denser clouds of stored traces accumulated through experience. A newly heard token of a high-frequency word therefore has relatively little weight against the large set of existing exemplars, whereas a newly heard token of a low-frequency word may exert a stronger influence on the representation activated for subsequent production. On this view, low-frequency words are less entrenched and may therefore be more susceptible to short-term influence from recently perceived tokens.

### 2.2 Behavioural task and accommodation

Task context has been identified as an important source of variation in phonetic accommodation. Accommodation has been investigated across a continuum of experimental settings, ranging from relatively non-interactive tasks such as imitation, shadowing, picture naming, and word matching to more interactive tasks such as picture matching, map tasks, mock interviews, and conversation (Pardo et al., 2022). These tasks differ in the immediacy of the relation between perceived input and subsequent production, the degree to which the input itself provides a direct phonetic target, and the extent to which speakers must plan, monitor, or socially evaluate their responses.

Previous work has shown that such task differences can affect both the degree and the direction of accommodation. In a direct comparison of speech shadowing and conversational interaction, Pardo et al. (2018) found comparable average levels of convergence across the two settings, but only weak consistency at the level of individual talkers: speakers who converged strongly in shadowing were not necessarily the same speakers who converged strongly in conversation. Increased cognitive load has also been shown to reduce perceived linguistic convergence (Abel and Babel, 2017). In more socially marked settings, task context may even favour divergence: Earnshaw (2021) found divergence in a mock police interview task, characterised by formality and asymmetric power relations, compared with more informal, symmetric conversational settings.

This variation across tasks is therefore theoretically important because different task contexts may favour different strengths or mechanisms of accommodation. In the present study, task type is not treated as a direct proxy for a single mechanism. Both shadowing and interaction may involve relatively automatic as well as more controlled processes. Rather, the task contrast is used to manipulate the immediacy and specificity of the relation between a perceived input and the speaker's subsequent output. We use *input–output coupling* to refer to this relation. It has three relevant components: the temporal proximity between input and output, the degree of form similarity or overlap between the perceived and produced material, and the amount and type of intervening processing required before production.

Input–output coupling is expected to be strongest when the perceived and produced forms are temporally adjacent, when they are identical or highly overlapping, and when the mapping from input to output requires minimal intervening processing. Shadowing tasks, which require immediate repetition of recently heard material, therefore provide a context of relatively strong input–output coupling. In such tasks, the perceived form supplies both the linguistic content and a direct phonetic target for the participant's response. The interaction task used here, although more interactive than shadowing, involved pre-recorded stimuli rather than live dialogue. It is therefore not treated as a fully naturalistic test of social interaction. Its relevance lies instead in the fact that it weakens input–output coupling along more than one dimension: participants must comprehend the preceding answer and question, select an appropriate lexical item, plan a response, and generalise the pronunciation pattern across different words sharing the same suffix.

### 2.3 Lexical frequency, phonetic realisation, and entrenchment

Lexical frequency is associated with a wide range of phonetic and processing effects at various levels of linguistic structure. High-frequency words and collocations are often produced more rapidly and with greater phonetic reduction than less frequent ones (Bybee and Scheibman, 1999; Jurafsky et al., 2001). For example, high-frequency regular past-tense verb forms in English have shorter word and *-ed* suffix durations than matched low-frequency forms (Losiewicz, 1992). High-frequency syllables are produced faster (Carreiras and Perea, 2004; Cholin et al., 2006) and with greater coarticulation (Benner et al., 2007; Croot and Rastle, 2004; Whiteside and Varley, 1998). Such syllable-level frequency effects have been interpreted within representational frameworks in which frequently used syllables are produced through the retrieval of articulatory routines stored in a mental syllabary (Levelt, 2000; Levelt and Wheeldon, 1994).

The present study approaches the relationship between lexical frequency and accommodation in terms of representational entrenchment. In usage-based phonology, linguistic experience shapes storage and access: repeated use strengthens representations, making high-frequency forms easier to access and more stable as stored routines (Bybee, 2001). High token frequency can have apparently opposing consequences: frequent forms may undergo reductive phonetic change earlier because they are produced more often and are therefore more often subject to the articulatory pressures associated with repeated use; at the same time, high token frequency strengthens or stabilises lexical representations, making frequent forms more resistant to restructuring or analogical change (Bybee, 2001). Following usage-based and exemplar-theoretic approaches, we use *entrenchment* to refer to the strength, stability, and routinisation of stored linguistic representations as a function of accumulated experience (Blumenthal-Dramé, 2017; Bybee, 2001; Pierrehumbert, 2001). Applied to accommodation, an entrenchment-based account predicts that high-frequency words, being supported by denser and more stable stored representations, should be less affected by a newly perceived token than low-frequency words, whose representations are less densely supported.

Todd et al. (2019) provide a more explicit exemplar-based formulation of this frequency effect. The model is concerned with diachronic sound change rather than short-term adaptation, but it formalises how lexical frequency can affect phonetic updating in a production–perception loop. In the model, individual word types are associated with distributions of stored exemplars, while phonological categories support generalisation across words. Production draws on these stored exemplars, while perception filters incoming tokens for storage: once a token has been identified as belonging to a particular word type and category, the relevant exemplar distribution is updated only if the token is sufficiently typical of that category and sufficiently discriminable from neighbouring categories; tokens that fail these evaluations are not stored. Lexical frequency enters the model through differences between high- and low-frequency word types in the density and robustness of their exemplar distributions. As a result, a newly encountered token does not have the same representational consequences for all words: its effect depends on the existing distribution of stored exemplars for that word and on the relation between the incoming token and neighbouring phonetic categories.

### 2.4 Lexical frequency and accommodation

Building on the entrenchment account, we propose that the extent to which the predicted frequency asymmetry is expressed in production depends on input–output coupling. When the same word is produced immediately after it is heard, the word-specific representation activated during perception is also directly relevant to the intended production, allowing the recent token's phonetic detail to influence the response. For delayed production of the same word, Goldinger's episodic account models retention as a feedback loop in which the representation maintained for the response is progressively reshaped by stored lexical traces, attenuating token-specific phonetic detail and shifting it towards the central tendency of the lexical category (Goldinger, 1998). Lexical identity introduces a further constraint: when the subsequently produced word differs from the perceived word, the production target draws on a different word-specific distribution, so the influence of the heard variant must instead generalise through the phonological and morphological material the words share, here the sub-lexical representation of the *-ig* suffix (Pierrehumbert, 2001; Todd et al., 2019). Temporal proximity and lexical identity therefore allow the recent token's word-specific influence to be expressed more directly in production. Accordingly, because recent tokens are predicted to have greater relative influence on low- than on high-frequency word representations, strong input–output coupling should make this frequency asymmetry more pronounced in production.

Empirical findings relevant to this prediction, however, are not straightforward. Goldinger's shadowing experiments provided evidence that speakers imitate phonetic details of recently heard speech even without explicit instructions to do so, and that such imitation was stronger for lower-frequency words and for items that had been repeated more often (Goldinger, 1998). These findings provide empirical support for linking lexical frequency and episodic memory to accommodation under conditions of strong input–output coupling.

Goldinger and Azuma (2004) provide further evidence that such effects are not limited to immediate shadowing. In their study, participants first read printed words aloud, were then exposed to auditory tokens of those words, and later read the same printed words again. Perceptual judges detected post-exposure imitation in the later readings, especially for lower-frequency words and for items with more training exposures. This finding is important because it suggests that episodic traces of heard words can influence later production even when speakers are not directly shadowing an auditory stimulus. At the same time, the effect was still word-specific: the previously heard and subsequently produced forms corresponded to the same lexical item.

However, later research suggests that frequency effects in phonetic convergence are variable rather than general. In a large-scale shadowing study using multiple model talkers, multiple shadowers, and both holistic and acoustic measures, Pardo et al. (2017) found no reliable main effect of word frequency on convergence. Instead, convergence was variable and inconsistent across measures: it was detected in the holistic AXB perceptual-similarity task and in some acoustic dimensions, but not uniformly across individual acoustic measures. Lexical effects also interacted with participant gender: female shadowers showed greater convergence to low-frequency than to high-frequency words, whereas male shadowers showed no comparable frequency effect. These findings suggest that frequency effects are not a uniform property of phonetic convergence, but depend on participant-related and other interacting factors.

More recent work further complicates the interpretation of frequency effects in convergence. Sanker (2021) examined participants' first and second readings of the same set of words in the absence of exposure to another speaker. When first-to-second changes were measured relative to another speaker's productions used as reference values, second productions could be classified as convergence, with a tendency towards greater apparent convergence for lower-frequency words. Sanker attributes this pattern to first-mention effects: initial productions of low-frequency words may be especially prone to hyperarticulation or disfluency, while later productions become more natural. Frequency-conditioned differences between an initial baseline and a later production may therefore partly reflect changes associated with producing the same word again rather than adaptation to another speaker. At the same time, Wade et al. (2023) show that frequency effects can also occur in expectation-driven convergence, where speakers shift towards a socially expected variant even though the specific target words are not produced by the model talker. Together, these findings indicate that frequency effects in accommodation may arise from several sources, including episodic-memory dynamics, repeated-production effects, lexical accessibility, and socially or contextually generated expectations.

The present study therefore treats lexical frequency as a factor whose influence may depend on task-related and social dimensions rather than as a uniform determinant of accommodation. Among the possible mechanisms discussed above, the present predictions centre on representational entrenchment and episodic-memory dynamics. On this account, frequency effects should be clearest when input and output are temporally adjacent and lexically identical. When different lexical items are involved, accommodation may still occur through shared sub-lexical material, but frequency-related differences in word-specific representations should be less directly reflected in production.

### 2.5 Gender and accommodation

Gender has been widely investigated in accommodation research, but findings remain inconsistent. Namy et al. (2002) reported greater accommodation in female than in male speakers, a result often cited as evidence for gender-based differences in sensitivity to phonetic detail and interpersonal alignment. However, this interpretation requires caution. Pardo et al. (2017) found no reliable main effect of talker sex on convergence. Instead, the clearest gender-related pattern concerned lexical properties: female shadowers showed stronger convergence to low-frequency than to high-frequency words, whereas male shadowers showed no comparable frequency effect. Pardo et al. (2017) therefore argue against a simple interpretation in which female speakers generally converge more than male speakers, and instead suggest that gender may condition the circumstances under which lexical properties affect convergence.

More broadly, the literature presents a rather heterogeneous picture. Some studies report stronger same-gender convergence (Lelong and Bailly, 2011), others stronger male–male convergence (Pardo, 2006; Thomason et al., 2013), and still others enhanced convergence in mixed-gender interactions (Levitan et al., 2012), while some find no significant gender effects (Kawasaki et al., 2013). This variability is also evident in shadowing studies. Pardo et al. (2013), using multiple model talkers and balanced female and male shadowers, found overall convergence but no modulation by talker sex or lexical properties, whereas Babel et al. (2014) found that female shadowers were more susceptible to differences in vocal attractiveness and gender typicality of individual model talkers. Taken together, these findings support the view that gender-related effects in accommodation are not reducible to a single main effect of talker gender, but may depend on task type, interlocutor pairing, lexical properties, model-talker characteristics, and the social meaning of the variable under investigation.

A broader sociolinguistic perspective leads to the same caution against treating gender as a simple main-effect predictor. Labov (2001) formulates the gender paradox in terms of women's greater conformity to overtly prescribed or prestigious linguistic norms, alongside their leadership in many changes from below. However, as Eckert (2000) argues, gender effects in variation should not be treated as stable properties of female and male speakers, but as locally constructed patterns that depend on the social meaning of the variable, the community, and the interactional setting. This point is important for the present study because the two main *-ig* variants do not differ only phonetically. As discussed in Section 2.6, the plosive variant may be interpreted as standard-oriented or orthographically influenced in the Saarland context, whereas fricative realisations have a more complex relation to both codified Standard German and local dialect. Gender effects in accommodation may therefore depend not solely on participant gender, but also on the direction of accommodation, which in turn may be related to the social interpretation of the target variant.

The present study consequently treats gender as a potential modulating factor rather than as a source of a uniform main-effect prediction. Following Pardo et al. (2017), particular attention is paid to whether participant gender modulates frequency-conditioned accommodation, and—additionally—to whether observed effects are sustained across tasks. At the same time, we examine whether gender effects are sensitive to the direction of accommodation, given the different regional, standard-language, and orthographic associations of the fricative and plosive variants. Any gender-related interpretation must remain cautious, however, because the participant sample in this study is not balanced by gender and because the distribution of participants' baseline pronunciation variants is uneven.

### 2.6 The *-ig* variable

The German adjectival suffix *-ig* provides a particularly useful test case for phonetic accommodation because it exhibits salient segmental variation without a change in lexical meaning. In Standard German pronunciation, the suffix is traditionally realised with a fricative, usually [ɪç], and this pronunciation is codified in pronunciation dictionaries (Kleiner and Knöbl, 2015). In actual usage, however, the suffix shows extensive regional and register-related variation. The final consonant may be realised as a fricative at a range of places of articulation, including [x], [ç], [ɕ], and [ʃ], or as a velar plosive [k] (Kleiner, 2010).

For the present study, the regional context is especially relevant. Most participants had attended school in Saarbrücken or the surrounding Saarland region. The local Rhine-Franconian dialect of Saarbrücken favours non-standard fricative realisations such as [ʃ] or [ɕ] rather than the codified Standard German [ç] variant (Braun and Mangold, 1984; Steitz, 1981). At the same time, the distribution and evaluation of the plosive variant are more complex than a simple regional contrast would suggest. The *Atlas zur deutschen Alltagssprache* reports that, although the older north–south distribution between [ç] and [k] pronunciations remains visible, [k] pronunciations are also reported in parts of the Rhenish, Hessian, and Palatine areas, where they may reflect hypercorrection in regions with [ʃ]-like fricative realisations (Elspaß and Möller, 2011). This pattern is also consistent with the role of orthography in encouraging the interpretation of [ɪk] as a standard-oriented form (Kehrein, 2012). In perception, Kehrein (2009) reports that *-ig* realised as [ɪk], although regionally distributed, is not judged as deviating from the standard. A plosive realisation in Saarland should therefore not be interpreted simply as an unexpected dialectal reflex, but may also reflect standard-oriented or orthographically influenced speech.

This complex sociophonetic status makes *-ig* especially suitable for the present study. The variable offers a clear phonetic contrast between fricative and plosive realisations, but the social and regional interpretation of these variants cannot be reduced to a simple opposition between standard and non-standard forms. In particular, a plosive realisation may reflect standard-oriented or orthographically influenced pronunciation rather than a straightforward regional variant, while fricative realisations themselves may differ in their relation to codified Standard German and local dialect. For this reason, the present study does not treat either variant as a default or generally expected pronunciation. Instead, the experimental design first establishes each participant's dominant pronunciation and then examines whether and under what conditions exposure to the alternative variant leads to accommodation.

### 2.7 The present study

Building on the theoretical and empirical background discussed above, the present study investigates how phonetic accommodation is shaped by the interaction of lexical frequency, behavioural task, and gender. While previous work has demonstrated that accommodation is influenced by both priming-based and socially mediated processes, and that lexical frequency is associated with phonetic realisation and representational entrenchment, not enough is known about how these dimensions interact. In particular, it remains unclear whether frequency effects in accommodation operate generally, or whether they are contingent on processing conditions and input–output relations.

The study addresses this question by examining speakers' production of the German adjectival suffix *-ig*. As discussed in Section 2.6, this variable is well suited to the study of accommodation because it involves a clear alternation between fricative and plosive realisations without a change in lexical meaning, while also showing substantial regional and standard-language variability. In the present design, each participant's baseline pronunciation was established experimentally before exposure to the alternative variant. Accommodation can therefore be operationalised as a shift towards the variant presented in the stimulus, i.e., away from the speaker's dominant baseline production.

Crucially, the design combines a manipulation of lexical frequency with two experimental tasks that differ in their processing demands and in the lexical relation between perceived input and subsequent production. The shadowing task involves immediate repetition of sentences containing the same target adjective that has just been heard. It therefore maximises input–output coupling while minimising message-level planning, providing conditions under which a recently perceived pronunciation variant is most directly available for subsequent production. In contrast, the interaction task requires participants to produce a different *-ig* adjective from the one presented in the preceding discourse. It therefore reduces direct lexical overlap between input and output and requires additional processing, including comprehension of the recorded answer and question, response selection, message planning, and monitoring of the task sequence. Although shadowing also involves perceptual, phonological, short-term memory, and articulatory demands, it provides a more direct and immediate mapping between perceived input and subsequent production. The task contrast is therefore interpreted not as a contrast between automatic and social mechanisms, nor as a simple difference in overall difficulty, but as a difference in the types of input–output mappings and processing demands that are most relevant to accommodation.

#### 2.7.1 Lexical frequency and accommodation

The first aim of this study is to determine whether lexical frequency influences phonetic accommodation. On an exemplar-based account, low-frequency items should be more susceptible to accommodation because they are less strongly entrenched: a newly perceived token carries greater relative weight within a sparser set of stored traces (Goldinger, 1998). However, we do not assume that this low-frequency advantage should operate as a uniform main effect. Rather, lexical frequency may interact with other factors that shape accommodation, including the input–output relation created by the task, gender, and the direction of accommodation.

#### 2.7.2 Task effects and processing demands

The second aim is to assess the role of behavioural task in shaping accommodation. Based on hybrid accounts of phonetic convergence, we expect accommodation to be stronger in shadowing than in interaction: the immediate input–output mapping and whole-word repetition in shadowing should favour the influence of the recently perceived variant, whereas the additional processing and across-word generalisation required in interaction may attenuate or reshape accommodation.

#### 2.7.3 Gender and social modulation

The third aim of the study is to examine whether accommodation is modulated by participant gender. Following Pardo et al. (2017), we do not treat participant gender as a predictor that should necessarily have a uniform effect across the experiment. Rather, we ask whether participant gender contributes to accommodation on its own or in interaction with task context, lexical frequency, and direction of accommodation. This allows gender to be treated as a factor whose effect may depend on the processing and sociolinguistic conditions under which accommodation takes place.

We also examine stimulus voice gender. Because the stimuli were pre-recorded and did not involve live interlocutor interaction, stimulus voice gender is not interpreted as a direct measure of interpersonal alignment; rather, it tests whether accommodation varies with model voice gender.

#### 2.7.4 Direction of accommodation

A fourth aim is to examine whether accommodation is affected by the direction of change. In the present design, participants with a dominant plosive baseline were exposed to fricative variants, whereas participants with a dominant fricative baseline were exposed to plosive variants. These two accommodation paths need not be equivalent. As discussed in Section 2.6, the fricative and plosive realisations of *-ig* differ not only phonetically, but also in their regional, standard-language, and orthographic associations. We do not formulate a strong directional prediction, because the distribution of baseline variants in the sample is uneven, but we include direction of accommodation as a potential moderating factor in order not to treat accommodation as necessarily symmetrical across the two variants.

#### 2.7.5 Exposure and adaptation over time

The fifth aim is to investigate whether accommodation changes with cumulative exposure to the alternative variant. Repeated exposure may increase the availability of that variant in the speaker's production system, leading to greater accommodation over the course of the experiment. From a usage-based and exemplar-theoretic perspective, such an effect would suggest that speakers' productions are shaped not only by the immediately preceding stimulus, but also by accumulating experience with the alternative pronunciation during the task.

#### 2.7.6 Implications for theories of accommodation

Finally, the study seeks to evaluate the extent to which the observed patterns support different theoretical accounts of accommodation. By comparing behaviour across tasks that differ in input–output coupling and in the type of processing demands they impose, and by examining the role of lexical frequency within these contexts, the study aims to contribute to hybrid models in which accommodation emerges from the interaction of the relation between perceived input and subsequent production, the task-specific processing demands, the representational strength of the forms involved, and speaker-related and social factors.

By combining controlled manipulation of lexical frequency with variation in task type, speaker and stimulus gender, and exposure, the present study provides a multifactorial perspective on phonetic accommodation. In doing so, it aims to clarify the conditions under which lexical frequency influences accommodation and to situate this effect within a processing-based account of adaptive speech behaviour.

## 3 Materials and methods

### 3.1 Participants

A total of 47 native speakers of German were recorded. Five participants were excluded before accommodation analysis. Four were excluded because their baseline productions were split approximately equally between the plosive and fricative *-ig* variants, and therefore did not provide a clear baseline preference. One further participant was excluded because of a recording failure: the baseline elicitation session was incomplete. All five excluded participants had taken part in the shadowing task; four of them had also participated in the interaction task and were therefore excluded from that task as well.

After exclusions, the analysed sample comprised 42 participants. The two tasks involved partially overlapping groups: 30 participants contributed data to the shadowing task (21 female, 9 male; mean age 25.57 years, SD = 2.49); 31 contributed data to the interaction task (20 female, 11 male; mean age 26.35 years, SD = 6.49); 19 took part in both tasks. Participants who took part in both tasks completed the interaction task at least 2 weeks after the shadowing task, in order to reduce potential carryover effects. Each participant completed the baseline elicitation only once. It took place immediately before shadowing for all 30 shadowing participants and immediately before interaction for the 12 participants who completed only that task. For the 19 participants who completed both tasks, the baseline collected before shadowing was therefore separated from the interaction session by at least 2 weeks and was used to define accommodation in both tasks.

For all retained participants, the dominant baseline variant accounted for at least 16 of 19 target-item productions (84.2%), providing a stable baseline relative to which accommodation could be defined. Across the 42 unique retained participants, 32 used the same variant in all their baseline productions, while the remaining 10 had one to three non-dominant realisations. Of these 10 participants, eight contributed data to the shadowing task and seven to the interaction task, with five contributing to both.

Participants completed a demographic questionnaire, in which they were asked to indicate their age, gender, and places where they had attended primary, lower secondary, and upper secondary school.^1^

### 3.2 Materials

The study focuses on the German adjectival suffix *-ig*, which varies between fricative and plosive realisations, as discussed in Section 2.6. The experiment included 19 target items (10 high-frequency and 9 low-frequency adjectives ending in *-ig*),^2^ as well as 60 filler items (also balanced for frequency) intended to reduce the predictability of experimental goals. High-frequency and low-frequency target items were sourced from the top and bottom thirds of the log_10_ probability range of *-ig* adjectives within the deWaC corpus, a web-crawled corpus of German containing 1.5 billion running words in a diverse range of genres, from newspaper articles to chat messages (Baroni et al., 2009). The high- and low-frequency categories were thus well separated by the middle third of the range, from which no items were included.

The stimuli used in the shadowing and interaction tasks were recordings of two phonetically trained native speakers of German (male and female), each producing both versions of the target adjectives. Recordings were checked for consistency and normalised for intensity.

### 3.3 Experimental tasks

The study consisted of three main components:

1. **Baseline elicitation**. Participants first produced the target and filler items in a controlled elicitation task. They were shown randomised questions on a screen, such as ‘What is a/another word for …?', along with partial orthographic cues that did not include the suffix itself (e.g. ‘r i c _ _ _ _' for *richtig*). This phase established each participant's default pronunciation variant.
2. **Shadowing task**. Participants listened to recorded sentences containing a target or filler item and immediately repeated each sentence. Target items were presented in the alternative pronunciation to each participant's baseline variant.
3. **Interaction task**. Participants first read a question out loud (*What is a word for …?*). A recorded voice answered (again, using the alternative pronunciation for target adjectives). Participants then heard a recorded question and gave a one-word answer. Target and filler items in each QA–QA pair were matched for frequency (either high or low).

In each accommodation task that they completed, participants produced all 19 target adjectives once, intermixed with the 60 filler items. Trial order was randomised separately for each recording session, so the sequential order predictor did not correspond to the same target items across participants and tasks. In the interaction task, each participant also heard all 19 target adjectives from the recorded interlocutor across the full set of QA–QA trials. The heard and produced target adjectives within a given QA–QA pair were always different and were matched for frequency, but because trial order was randomised, a given adjective could be produced before or after the participant had heard the same adjective as the recorded answer in another trial. When a target adjective occurred in the recorded answer in one stimulus voice gender, the recorded question eliciting that same adjective was produced by the other voice gender.

Although both shadowing and interaction involve exposure to and production of target items, the two tasks differ in the relation between perceived input and subsequent production. In the shadowing task, participants repeated the sentence they had just heard, including the same target adjective. Shadowing therefore involved identity between the perceived and produced target item, allowing whole-word priming and maximising input–output coupling. In the interaction task, by contrast, participants heard one *-ig* adjective in the recorded answer and subsequently produced a different *-ig* adjective as their own answer. The adjective pairs were matched for frequency, but did not involve repetition of the same lexical item. Accommodation in this task therefore required the perceived pronunciation variant to generalise across different words sharing the same suffix. Beyond this difference in input–output mapping, the tasks also differed in broader processing demands. Shadowing involved immediate repetition and relatively little message-level planning, although it still required perception, short-term retention, phonological encoding, and articulation. The interaction task involved comprehension of the recorded answer and question, response selection, message planning, and monitoring of the QA–QA sequence. Thus, the contrast between the two tasks is not simply a contrast between lower and higher overall difficulty, but a contrast between different input–output mappings and broader processing conditions relevant to accommodation.

The stimuli in the shadowing and interaction tasks contained an equal number of female and male voices and were presented in random order.

After performing the interaction task, participants also completed a self-report and attitudes questionnaire, indicating their preferred pronunciation and evaluating the acceptability of the alternative variant for each of the target adjectives. The questionnaire was administered in German, using the same fixed item order for all participants. For each adjective, participants were asked how they would pronounce it, *Wie würden Sie* <item> *aussprechen?* and selected either *mit “ich”* or *mit “ik”*. They then evaluated the alternative variant in response to *Die andere Version*, with the options *ist auch ok* (is also ok), *ist falsch* (is incorrect), or a free-response completion *ist* __________. The questionnaire was used as a supplementary descriptive measure of participants' self-reported pronunciation preferences and explicit evaluations of the alternative variants. The questionnaire was exploratory rather than part of the core experimental design. It was introduced together with the interaction-task recordings, and was therefore completed only by participants who took part in the interaction task; participants who took part only in the shadowing task did not complete it. In addition, because the questionnaire was administered immediately after the interaction task, responses may have been influenced by participants' recent exposure to the alternative variants in that task; the questionnaire therefore cannot determine whether attitudes predicted accommodation or were themselves influenced by prior exposure and accommodation behaviour.

### 3.4 Coding of pronunciation variants

Participants' productions of the target adjectives were coded for the realisation of the final consonant of the *-ig* suffix, distinguishing two pronunciation variants: a fricative and a plosive realisation. The phonetic contrast was treated as a categorical variant alternation because it involves a clear difference in manner of articulation, without a change in lexical meaning. The analysis therefore focuses on whether participants adopted the alternative suffix variant presented in the stimulus, rather than on gradient acoustic convergence within a variant category.

The participants' responses were annotated perceptually by a native speaker of German with training in phonetics. The annotator listened to the productions and labelled each target response as fricative or plosive. The annotator was instructed to flag cases in which the classification was uncertain, but no such cases were reported. The four authors subsequently checked a random sample of approximately 20% of the annotations and found no disagreements with the original coding.

**Figure 1:**
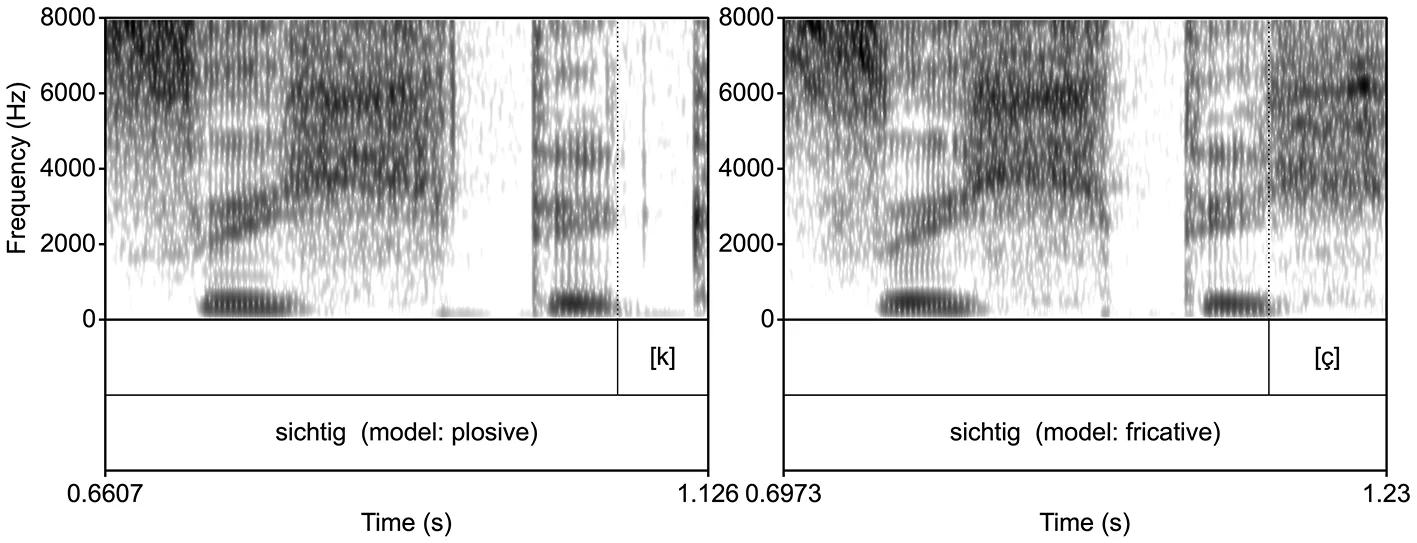
Representative spectrograms of a model speaker's plosive (left) and fricative (right) productions of the adjective *sichtig*.

**Figure 2:**
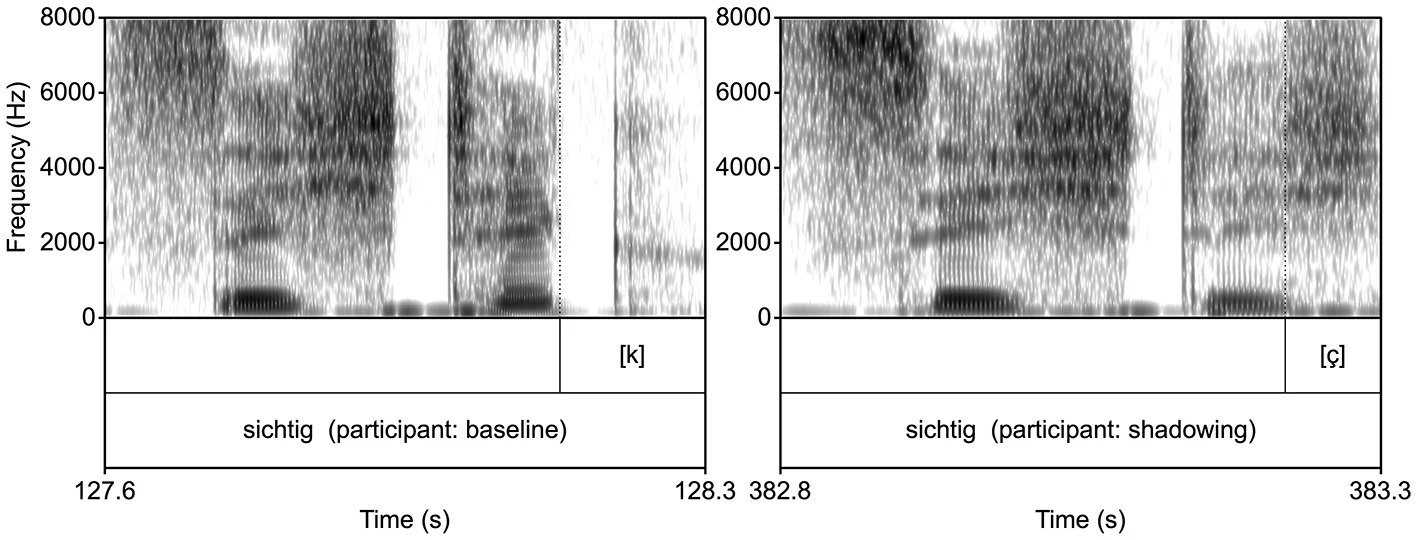
Representative spectrograms of a participant's productions of the adjective *sichtig*: baseline plosive realisation (left) and fricative realisation in the shadowing task (right).

Representative spectrograms are shown in Figures 1, 2. Figure 1 illustrates the contrast in the female model speaker's productions of the same adjective with plosive and fricative *-ig* realisations. Figure 2 illustrates the categorical shift in a female participant's productions, showing a baseline plosive production and a subsequent fricative production in the shadowing task. These examples are included to make the basis for the categorical coding transparent.

### 3.5 Statistical analysis

Across the shadowing and interaction tasks, 1,159 target-item responses were recorded (30 × 19 + 31 × 19). Twenty-one individual responses were excluded due to technical recording issues, prolonged hesitations or abandoned responses, or failure to produce the intended answer in the interaction task. The final dataset therefore comprised 1,138 analysable responses. The excluded responses were not concentrated in any particular participants or target adjectives.

Accommodation was encoded as a binary outcome variable: TRUE if the participant's production of a target adjective matched the alternative variant presented in the stimulus, and FALSE if the participant retained their dominant baseline pronunciation. For the minority of participants whose baseline productions were not fully consistent, an occasional response coded as accommodation may therefore reflect their sporadic baseline variation rather than adaptation to the stimulus, introducing a small amount of noise into the trial-level measure.

Accommodation was analysed using generalised linear mixed-effects models (GLMMs) with a binomial error distribution and a logit link function. Model selection was based on likelihood-ratio comparisons, AIC/BIC, and theoretical interpretability. We first considered the experimental predictors task, lexical frequency, participant gender, stimulus voice gender, direction of accommodation, and order. Sequential order was *z*-standardised before modelling. Because each participant contributed multiple observations, participant was included as a random intercept. A random intercept for target adjective (item) was evaluated but was not retained because it did not improve model fit. Stimulus voice gender was also not included in the final model after evaluation.

The final model retained sequential order as a main-effect covariate, the theoretically central interaction between task, lexical frequency, and participant gender, as well as direction together with its interactions with task and participant gender. Interactions between order and the other predictors were tested in a sensitivity model but were not retained, since they did not improve model fit. The final model structure is given in Equation 1.


$$\begin{array}{c} \text{accommodation}\sim \text{order}+\text{task}*\text{frequency}*\text{gender}\\ +\text{direction}+\text{task }:\text{ direction}+\text{gender }:\text{ direction}\\ +\left(1|\text{participant}\right) \end{array}$$ (1)

Statistical analyses were conducted and related graphs were produced in R (R Core Team, 2024); GLMMs were fitted using package *lme4* (Bates et al., 2015). Spectrograms were created in Praat (Boersma and Weenink, 2024).

## 4 Results

The vast majority of participants demonstrated a consistent baseline preference for the plosive pronunciation (76.7% of participants in shadowing and 74.2% in interaction). As discussed in Section 2.6, this pattern should not be interpreted simply as a regional feature, since most participants came from Saarbrücken or the surrounding Saarland region, where local dialectal fricative realisations differ from the codified Standard German fricative and where the plosive variant may represent standard-oriented speech. Most participants were therefore exposed to recordings of the fricative pronunciation of target adjectives. Table 1 shows the distribution of dominant baseline variants by participant gender and task. This breakdown is relevant for interpreting gender-related effects because the cross-classification of participant gender and direction of accommodation is uneven in the analysed sample: the male and fricative-to-plosive subsets are smaller than the female and plosive-to-fricative subsets, respectively. Effects involving participant gender and direction should therefore be interpreted as conditional patterns within an unevenly distributed sample, rather than as stable gender and direction differences in accommodation. As an additional descriptive check, all target adjectives showed broadly similar baseline distributions: fricative realisations accounted for approximately 20–30% of elicited baseline productions for every target item.

**Table 1:** Dominant baseline variant by participant gender and task.

Task	Participant gender	P → F	F → P	Total
Shadowing	Female	17	4	21
	Male	6	3	9
	Total	23	7	30
Interaction	Female	16	4	20
	Male	7	4	11
	Total	23	8	31

Plosive-baseline participants were exposed to the fricative variant in the accommodation tasks (P → F), whereas fricative-baseline participants were exposed to the plosive variant (F → P).

In descriptive terms, accommodation occurred in 301 of 563 analysed shadowing trials (53.5%), while the dominant baseline variant was retained in the remaining 262 trials (46.5%). In the interaction task, accommodation occurred in 157 of 575 analysed trials (27.3%), meaning that the dominant baseline variant was retained in 418 trials (72.7%). Overall, accommodation occurred in 458 of 1138 analysed trials (40.2%).

We next report the GLMM model-selection procedure, followed by the fixed-effect estimates of the final model. Model selection started from a main-effects baseline model containing order, task, lexical frequency, participant gender, stimulus voice gender, direction of accommodation, and a random intercept for participant (M0 in Table 2). A full two-way interaction model substantially improved the fit over this baseline (M1). The full two-way model was then reduced by removing unsupported interactions, yielding a more parsimonious model with lower AIC and BIC (M2). Because the two directions of accommodation may not be equivalent, we specifically evaluated whether the frequency effect differed by direction. The full two-way model included the frequency:direction interaction, but single-term deletion showed that it was not supported, χ^2^(1) = 0.63, *p* = 0.4277. It was therefore excluded from the reduced model. Next, removing stimulus voice gender from the reduced two-way structure did not worsen the fit and further improved both AIC and BIC, so voice was not retained (M3).

**Table 2:** Model-selection summary for the binomial mixed-effects analysis of accommodation.

Model	Model structure	df	AIC	BIC	Comparison	χ^2^	*p*
M0	order + task + frequency + gender + voice + direction + (1|participant)	8	898.39	938.69	—	—	—
M1	order + (task + frequency + gender + voice + direction)^2^ + (1|participant)	18	848.36	939.03	M1 vs. M0	70.03	0.0000
M2	order + task * frequency + task * gender + frequency * gender + voice + direction + task:direction + gender:direction + (1|participant)	13	841.93	907.41	M2 vs. M1	3.56	0.6141
M3	order + task * frequency + task * gender + frequency * gender + direction + task:direction + gender:direction + (1|participant)	12	840.23	900.68	M3 vs. M2	0.31	0.5797
**M4**	order + task * frequency * gender + direction + task:direction + gender:direction + (1|participant)	13	835.41	900.89	M4 vs. M3	6.82	0.0090
M5	M4 + voice	14	837.13	907.65	M5 vs. M4	0.28	0.6000
M6	M4 + (1|item)	14	836.77	907.28	M6 vs. M4	0.64	0.4229
M7	M4 + order interactions with task, frequency, gender, and direction	17	839.27	924.90	M7 vs. M4	4.14	0.3878
M8	order + (task + frequency + gender + direction)^3^ + (1|participant)	17	835.50	921.13	M8 vs. M4	7.91	0.0950
M9	M4 + task:gender:direction	14	829.93	900.45	M9 vs. M4	7.48	0.0062

*P*-values are likelihood-ratio tests for the model pair indicated in the Comparison column. Selected final model: M4.

The theoretically central task × frequency × gender interaction was then added to the reduced no-voice model (M4). This significantly improved the fit, χ^2^(1) = 6.82, *p* = 0.0090, and reduced AIC, while BIC remained virtually unchanged. This model was therefore selected as the final model. Additional checks showed that reintroducing stimulus voice gender (M5), a random intercept for target item (M6), interactions between order and the main predictors (M7), or a broader three-way interaction structure (M8) did not provide sufficient improvement. Although M9 improved the likelihood and AIC, and produced a marginally lower BIC, it was not retained for inference because the model diagnostics indicated separation-related instability. Specifically, the model produced very large standard errors and convergence warnings indicating a degenerate Hessian. Model selection therefore proceeded among diagnostically stable models, within which M4 provided the best-supported structure.

The fixed-effect estimates from the selected final model are reported in Table 3 and summarised below. The reference level for the model is the *interaction* task, *high* frequency, *female* participant, and *plosive-to-fricative* (P → F) direction of accommodation.

- The model showed a significant positive effect of order (β = 0.47, *p* < 0.0001), indicating that accommodation increased as exposure to the alternative variant accumulated over the course of the experiment.
- Since interaction is the reference level for task, the positive coefficient for task indicates that (for high-frequency items produced by female participants in the plosive-to-fricative direction) accommodation was significantly more likely in shadowing than in interaction (β = 1.77, *p* = 0.0001).
- Lexical frequency did not have a significant main effect (*p* = 0.2882), providing no reliable evidence of a frequency effect in the interaction task for female participants with a plosive-to-fricative direction. However, the positive task:frequency interaction (β = 1.68, *p* = 0.0012) indicates that, for female participants, the low- vs. high-frequency contrast was larger in shadowing than in the interaction task; the model therefore estimates greater accommodation for low-frequency than for high-frequency items in shadowing. The significant task:frequency:gender interaction (β = 2.60, *p* = 0.0115) indicates that this task-dependent difference in the frequency effect was larger still for male participants.
- Participant gender had a significant main effect: the negative coefficient for male participants indicates that, in the interaction task with high-frequency items in the plosive-to-fricative direction, male participants showed significantly less accommodation than female participants (β = −4.02, *p* = 0.0030). The positive task:gender interaction (β = 1.86, *p* = 0.0388) indicates that this gender difference was reduced in shadowing relative to the interaction task. The non-significant frequency:gender interaction (*p* = 0.0901) provides no strong evidence that the gender effect differed by frequency within the interaction task alone; however, as noted above, the significant task:frequency:gender interaction shows that gender did modulate the task-dependent frequency effect.
- The main effect of direction was not significant (*p* = 0.1567), but direction interacted significantly with both task and participant gender. The negative task:direction interaction (β = −3.92, *p* = 0.0000) shows that the task effect differed depending on whether participants were exposed to the fricative or plosive variant. The negative gender:direction interaction (β = −5.75, *p* = 0.0323) indicates that the effect of accommodation direction differed by participant gender. Because most participants had a plosive baseline and were therefore exposed to fricative stimuli, these direction effects should be interpreted cautiously; nonetheless, they show that accommodation was not symmetrical across the two directions of shift.

**Table 3:** Results of the selected final GLMM, accommodation ~ order + task * frequency * gender + direction + task:direction + gender:direction + (1|participant).

Fixed effect	β	SE	*z*	*p*
(Intercept)	−2.13	0.68	−3.14	0.0017
order	0.47	0.10	4.67	0.0000
task [Shadowing]	1.77	0.45	3.95	0.0001
frequency [Low]	0.27	0.25	1.06	0.2882
gender [M]	−4.02	1.35	−2.97	0.0030
direction [F → P]	−1.87	1.32	−1.42	0.1567
task [Shadowing] : frequency [Low]	1.68	0.52	3.25	0.0012
task [Shadowing] : gender [M]	1.86	0.90	2.07	0.0388
frequency [Low] : gender [M]	−0.86	0.51	−1.70	0.0901
task [Shadowing] : direction [F → P]	−3.92	0.58	−6.73	0.0000
gender [M] : direction [F → P]	−5.75	2.68	−2.14	0.0323
task [Shadowing] : frequency [Low] : gender [M]	2.60	1.03	2.53	0.0115

Intercept: log-odds of the outcome for the *interaction* task, *high* frequency, *female* participant gender, and *plosive-to-fricative* (P → F) direction of accommodation. Marginal *R*^2^ = 0.349; conditional *R*^2^ = 0.826.

**Figure 3:**
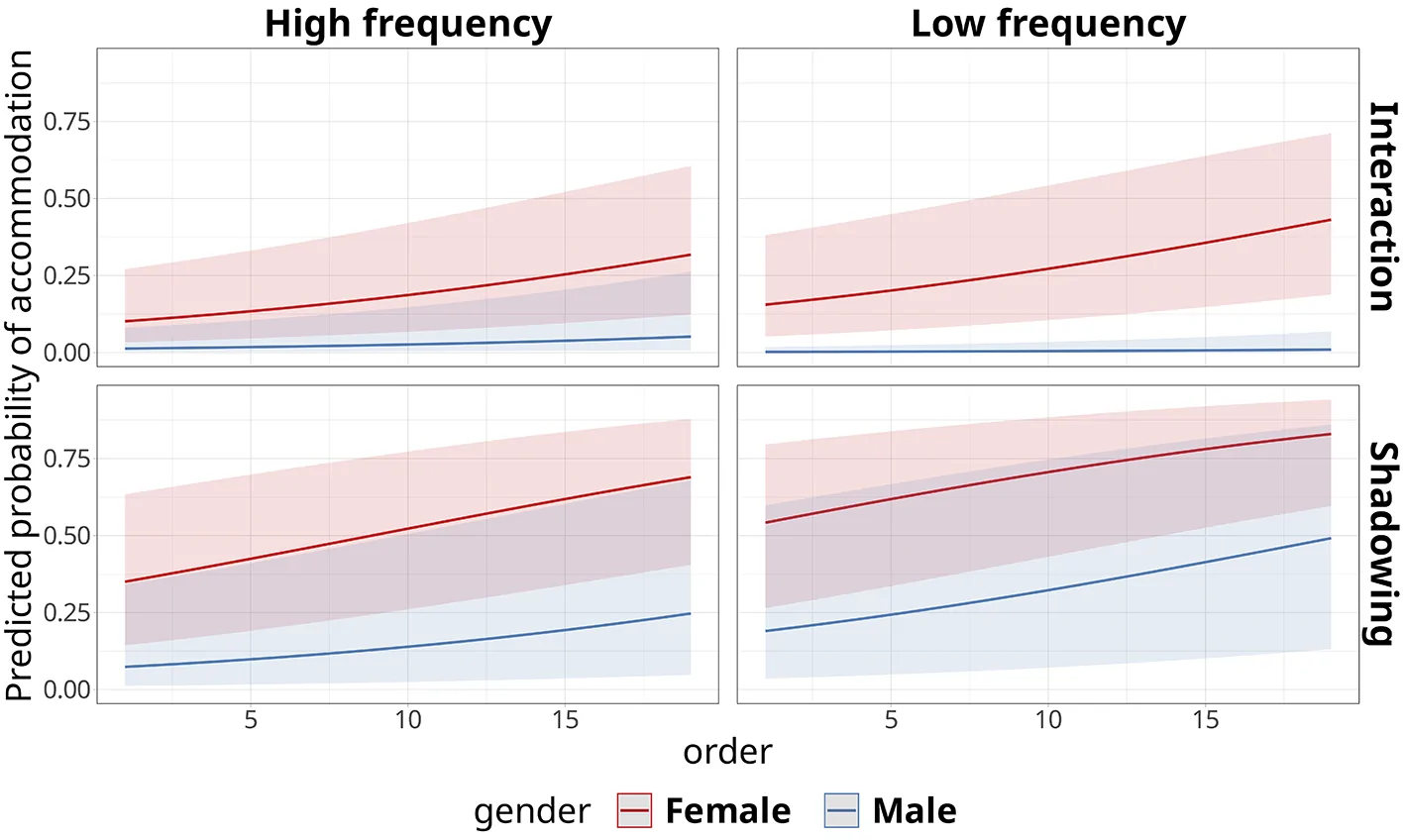
Relation of accommodation to sequential order, participant gender, lexical frequency, and task type; 95% CIs.

The main patterns described above are shown in Figure 3.

Participants' written responses in the supplementary self-report and attitudes questionnaire were examined as an exploratory descriptive supplement, focusing on how closely self-reported pronunciation preferences matched baseline productions and how participants evaluated the alternative *-ig* variants. As noted in Section 3.3, the questionnaire was completed only by participants who took part in the interaction task and was administered immediately after that task. It therefore cannot provide an independent test of participants' pronunciation awareness, their attitudes towards the variants, or the relation of either to accommodation behaviour. Most self-reported pronunciation choices aligned with participants' baseline productions: 74.6% of all responses matched the participants' baseline pronunciations, and 77.4% of participants showed more than 50% agreement between self-reported and baseline behaviour.

For attitudes towards the alternative variants, 82.1% of all responses indicated that the other pronunciation was ‘also OK', 14.8% that it was ‘incorrect', and the remaining 3.1% consisted of participants' own evaluations, mostly that the alternative sounded ‘odd' or ‘unusual'. When broken down by lexical frequency, positive responses (‘also OK') were more frequent for alternative pronunciations of high-frequency items (86.5%) than for alternative pronunciations of low-frequency items (77.3%). When broken down by direction of accommodation, positive responses were given in 81.5% of cases in the plosive-to-fricative direction and in 84.7% of cases in the fricative-to-plosive direction. By participant gender, positive responses were given in 81.0% of female participants' responses and 84.6% of male participants' responses. A further exploratory GLMM predicting positive attitudes towards the alternative pronunciation from frequency, participant gender, and accommodation direction showed significant effects of frequency (*p* = 0.0249), direction (*p* = 0.0073), and their interaction (*p* = 0.0191), but no significant effect of gender (*p* = 0.4595). This pattern suggests that attitudes towards the alternative pronunciation may vary by lexical frequency and direction of accommodation. However, these findings are treated as tentative rather than conclusive and are not used as a primary basis for interpreting accommodation behaviour.

## 5 Discussion

The present study set out to examine how phonetic accommodation is jointly shaped by lexical frequency, behavioural task, gender, exposure, and direction of accommodation, using variation in the German adjectival suffix *-ig* as a test case. The results reveal a systematic but non-uniform pattern. Accommodation was more frequent in shadowing than in interaction, increased over the course of the experiment, and varied with participant gender and direction of change. Lexical frequency did not exert a uniform main effect: instead, low-frequency items elicited more accommodation than high-frequency items specifically in shadowing, and this task-dependent frequency effect was further modulated by participant gender. By contrast, stimulus voice gender did not significantly affect accommodation. Taken together, these findings support a view of accommodation as a multifactorial and context-sensitive phenomenon, shaped by the interaction of input–output coupling, representational entrenchment, task-specific processing demands, and speaker-related factors. In what follows, we discuss the findings in relation to the aims stated in Section 2.7.

### 5.1 Lexical frequency and accommodation

The first aim of the study was to determine whether lexical frequency influences phonetic accommodation. The results provide partial but qualified support for the hypothesis that low-frequency items elicit more accommodation. Lexical frequency did not have a uniform main effect across the experiment. Instead, the frequency effect was conditional: low-frequency items elicited more accommodation than high-frequency items in shadowing, whereas no comparable effect emerged in the interaction task. This task-dependent pattern was further modulated by participant gender, with the difference between high- and low-frequency items changing more strongly across tasks for male than for female participants. This finding is broadly consistent with Goldinger's observation that low-frequency items can elicit stronger imitation in shadowing (Goldinger, 1998), while also aligning with later evidence that frequency effects in phonetic convergence are variable rather than general (Pardo et al., 2017).

The asymmetry between high- and low-frequency items in shadowing is compatible with usage-based and exemplar-theoretic accounts in which repeated experience strengthens word-specific representations, while recently perceived tokens may update or temporarily increase the activation of those representations (Bybee, 2001; Goldinger, 1998; Todd et al., 2019). High-frequency words are supported by denser sets of stored traces, so the influence of a newly heard token is diluted by prior experience; for low-frequency words, a new token carries greater relative weight and may therefore exert a stronger influence on subsequent production. In the present study, the alternative *-ig* variant heard in shadowing may therefore have had greater representational impact for low-frequency adjectives than for high-frequency ones.

The mechanism proposed in Section 2.4 predicts that shadowing allows a just-heard token of a low-frequency word to exert its relatively strong influence more readily on production because the token is recent and corresponds to the intended lexical item. The interaction task retains suffix-level overlap, so the perceived variant can still activate or update a representation relevant to *-ig*. However, because the response is a different adjective, the influence of the word-specific trace must generalise across lexical distributions and competes less directly with the stored exemplars of the response word. This does not mean that phonetic convergence cannot generalise beyond the specific heard item: convergence can extend to unheard material in some domains, for example in VOT imitation (Nielsen, 2011). Rather, the proposed mechanism explains why a low-frequency advantage could be more visible in shadowing without assuming that accommodation is impossible across words. More generally, frequency-based differences in representational strength should be most visible when the task provides a sufficiently direct relation between the recently perceived token and the speaker's subsequent production.

A methodological qualification should be made regarding repeated production across experimental phases. As discussed in Section 2.4, frequency-conditioned differences between initial and later productions may arise when speakers produce the same words more than once, even without adaptation to another speaker (Sanker, 2021). This possibility is especially relevant to the shadowing task, because all participants produced the target adjectives during baseline elicitation and then produced them again shortly afterwards in shadowing. The timing differed for the interaction task: the 12 participants who completed only interaction produced the baseline forms immediately beforehand, whereas the 19 returning participants had last produced the target adjectives at least 2 weeks earlier. Repeated-production effects may therefore have been weaker for a substantial proportion of the interaction sample, which could have contributed to the frequency effect emerging in shadowing but not interaction. However, the contribution of repeated production to the frequency effect observed in shadowing cannot be separated from genuine accommodation without a control condition—effectively a repeated baseline-style elicitation—in which participants would produce the target words a second time without intervening auditory exposure to the alternative variant.

Another possibility is that frequency effects were more detectable in shadowing because overall accommodation was stronger in that task, as discussed in Section 5.2. When accommodation rates are lower, as in the interaction task, there is less opportunity for frequency-conditioned differences to emerge. The task-dependent frequency effect should therefore be interpreted as arising from a combination of factors: differing processing demands, stronger input–output coupling due to exact lexical repetition, and higher overall accommodation.

The exploratory questionnaire results raise a related possibility, but only tentatively. Positive attitudes towards the alternative pronunciation appeared to vary not only with lexical frequency, but also with direction of accommodation, suggesting that explicit evaluations of the alternative variant may depend on the particular accommodation path involved. However, because the questionnaire was not part of the core experimental design, was completed only by participants who took part in the interaction task, and was administered immediately after that task, these results cannot establish that attitudes shaped accommodation behaviour. We therefore treat the attitude results as a possible contextual consideration, rather than as an explanation for the absence of a frequency effect in interaction.

Overall, these findings indicate that lexical frequency shapes accommodation primarily when the task provides a relatively direct route from a recently perceived pronunciation variant to subsequent production.

### 5.2 Task effects and processing demands

The next aim was to assess the role of behavioural task in shaping accommodation. The results show a clear overall task difference: accommodation occurred in 53.5% of analysed shadowing trials, compared with 27.3% of analysed interaction trials. The GLMM also showed that, in the model's reference configuration (i.e., high-frequency items produced by female participants with a plosive-to-fricative direction), accommodation was significantly more likely in shadowing than in interaction. This finding provides evidence that accommodation is sensitive to the relation between perceived input and subsequent production, and to the processing demands imposed by the task.

Shadowing involves immediate repetition of recently heard material, with relatively little message-level planning. It therefore provides a task context in which input–output coupling is strong: the perceived form supplies both the linguistic content and an immediately available phonetic target for production, allowing the recently heard pronunciation variant to influence the participant's response with minimal intervening processing. This direct mapping is compatible with alignment-based accounts, in which perceived linguistic forms can activate corresponding representations in production (Pickering and Garrod, 2004, 2013, 2021). The repetition of the same target adjective also makes the shadowing task relevant to usage-based and exemplar-theoretic accounts, in which a recently heard token may activate or temporarily increase the availability of word-specific stored representations (Bybee, 2001; Goldinger, 1998; Todd et al., 2019).

The interaction task, on the other hand, introduced additional processing between perception and production. Participants had to process the recorded answer and question, select an appropriate lexical item, plan their response, and monitor the task sequence. In addition, as discussed above, the interaction task removed lexical-item identity between input and output: participants did not reproduce the same adjective, although the heard and produced adjectives shared the same *-ig* suffix. Any accommodation therefore had to generalise across different words sharing the same suffix. This absence of whole-word identity, despite suffix-level overlap, provided a further reason why accommodation was weaker in interaction than in shadowing.

At the same time, the task effect was not completely independent of the direction of accommodation. The significant task:direction interaction indicates that the difference between shadowing and interaction varied depending on whether participants were exposed to the fricative or plosive variant. This asymmetry is consistent with the sociophonetic status of the *-ig* variable discussed in Section 2.6: the two directions of accommodation do not necessarily involve equivalent sociolinguistic shifts. Because most participants had a plosive baseline and were therefore exposed to fricative stimuli, the overall task contrast is driven primarily by plosive-to-fricative accommodation. The direction interaction should therefore be interpreted with caution, but it suggests that task effects are shaped not only by processing demands and lexical overlap, but also by which pronunciation variant speakers are exposed to.

The task contrast therefore reflects differences in the immediacy and specificity of the mapping between perceived input and subsequent production. Accommodation was strongest when the mapping was immediate and the perceived and produced forms were lexically identical, as in shadowing. When production required response selection, message planning, task-sequence monitoring, and suffix-level generalisation across words, as in the interaction task, accommodation was attenuated, and the resulting production patterns may have been shaped more strongly by participants' established production preferences and, potentially, by direction-related asymmetries in the sample.

### 5.3 Gender and social modulation

The third aim was to examine the role of gender-related factors in accommodation. While women showed higher accommodation overall, participant gender also modulated how accommodation was shaped by task, lexical frequency, and direction of change. Stimulus voice gender, by contrast, had no detectable effect. These findings fit the heterogeneous picture in the broader literature reviewed in Section 2.5, where gender effects in accommodation vary with task, interlocutor pairing, model-talker characteristics, lexical properties, and social context (Babel et al., 2014; Lelong and Bailly, 2011; Levitan et al., 2012; Pardo, 2006; Pardo et al., 2013; Thomason et al., 2013).

The most directly relevant comparison is with Pardo et al. (2017), who found no reliable main effect of talker sex in a large-scale shadowing study but reported stronger convergence to low- than high-frequency words among female shadowers, with no comparable frequency effect among male shadowers. The present results align with the broader conclusion that gender can condition frequency-related accommodation, although the specific female–male pattern was not identical: here, frequency effects varied jointly with task and participant gender, as detailed below. The present study also differs in comparing shadowing with interaction, as well as in examining categorical variant selection rather than holistic and gradient acoustic convergence in word shadowing.

In the present data, the increase in accommodation from interaction to shadowing among female participants was accompanied by a low-frequency advantage. The significant task:frequency:gender interaction indicates that this relation between task and lexical frequency differed for male and female participants. Given the size of the male subset, however, this interaction should not be interpreted as a stable gender contrast in the population. It nevertheless supports the general conclusion that lexical-frequency effects in accommodation are conditional: they emerge most clearly in shadowing, but their strength is also shaped by participant-related factors.

Gender also interacted with accommodation direction. This finding is important because the two *-ig* variants differ in their regional, standard-language, and orthographic associations, as discussed in Section 2.6. A sociolinguistic interpretation is therefore possible. The direction-related gender pattern suggests that men may be less likely than women to accommodate from a fricative baseline towards the plosive variant. If the plosive realisation is interpreted locally as a standard-oriented or orthographically supported form, as suggested in Section 2.6, this would be compatible with Labov's (2001) observation that gender differences in variation may reflect differential orientation to prescribed or standard-associated variants. This interpretation must remain tentative, however, because, as shown in Table 1, both the fricative-to-plosive subset and the male subset are relatively small, the social meaning of *-ig* is locally complex, and neither the fricative nor the plosive variant can be treated as a simple prestige or non-prestige form across all contexts. As Eckert (2000) emphasises, gender effects in variation are locally constructed rather than stable properties of female and male speakers. The direction-related gender pattern should therefore be understood as evidence that accommodation may be sensitive to the sociophonetic status of the target variant, not as evidence for a general gender difference in accommodation towards either *-ig* realisation.

At the same time, accommodation in the present paradigm was not significantly affected by the gender of the stimulus voice. Stimulus voice gender was tested during model selection, but removing it did not worsen model fit and improved the information criteria; reintroducing it into the selected final model likewise did not improve the fit. We therefore find no evidence that participants accommodated differently to male and female recorded voices in this design. This null effect should be interpreted in relation to the experimental setting: the stimuli were pre-recorded, the task did not involve live interlocutor interaction, and potential stimulus-voice effects may have been outweighed by stronger predictors such as task, lexical frequency, participant gender, and direction of accommodation.

### 5.4 Exposure and adaptation over time

Another aim was to investigate the role of exposure in accommodation. The results show a clear effect of sequential order, with accommodation increasing over the course of the experiment. This indicates that accommodation was not merely an immediate response to individual stimuli, but also reflected cumulative exposure to the alternative pronunciation variant.

Such gradual strengthening of accommodation is compatible with usage- and exemplar-based perspectives, in which repeated exposure to a variant can increase its availability in the speaker's production system. On this view, each exposure to the alternative *-ig* realisation may temporarily activate, reinforce, or update the relevant representation, making that variant more likely to be produced in later trials.

Importantly, interactions between order and the other predictors were tested but not retained in the final model. The exposure effect should therefore be interpreted as a general increase in accommodation over the course of the experiment; there was no evidence that adaptation over time differed reliably by task, lexical frequency, participant gender, or direction of accommodation.

### 5.5 Implications for theories of accommodation

The final aim of the study was to evaluate the implications of the observed patterns for theoretical accounts of phonetic accommodation. The results support a hybrid view in which the influence of recently perceived speech forms depends on the representational status of the linguistic material involved, the task-specific processing conditions under which perception and production are linked, and speaker-related factors. Such a view is in line with approaches that treat accommodation as jointly shaped by automatic and socially mediated processes (Babel, 2010, 2012; Coles-Harris, 2017; Krauss and Pardo, 2004; Pardo, 2012), while also recognising that the present study provides stronger evidence for processing- and speaker-related modulation than for direct social-interaction effects.

Taken as a whole, the findings suggest that accommodation is shaped by three analytically distinguishable, but interacting, domains: the relation between perceived input and subsequent production, including task-specific processing demands, the representational strength of the forms involved, and speaker-related and social factors.

The first domain concerns what we have called input–output coupling. This is not a single property of a task, but a relation made up of several partly overlapping components. Accommodation should be strongest when the perceived and produced forms are close in time, when they are lexically identical or highly similar, and when little intervening processing is required before production. These components are not fully independent. Increasing the temporal distance between input and output, or reducing lexical identity to partial form overlap, may itself increase processing demands, because the speaker must retain, abstract, or generalise the perceived variant rather than simply repeat the same word. In the present study, shadowing maximised input–output coupling: the target word was heard and then immediately repeated. The interaction task weakened it in several ways: the produced adjective was different from the heard adjective, meaning that accommodation had to generalise across words via the shared *-ig* suffix; in addition, production involved comprehension of the recorded answer and question, response selection, message planning, and monitoring of the QA–QA sequence. The task effect observed here should therefore not be interpreted as a simple contrast between automatic and social mechanisms, or between easy and difficult tasks, but as evidence that the path from input to output matters for accommodation.

The second domain concerns the representational status of the linguistic forms themselves. On a usage-based and exemplar-theoretic account, high-frequency words are supported by denser and more stable stored representations, whereas low-frequency words have sparser representational support. A newly heard pronunciation variant should therefore carry greater relative weight for a low-frequency word than for a high-frequency one. The present results suggest, however, that this difference in representational modifiability is not sufficient on its own to produce a frequency effect on accommodation. Frequency effects became observable primarily in shadowing, where the recently heard and subsequently produced forms were temporally adjacent and lexically identical. When the task required suffix-level generalisation across different words, the frequency effect was attenuated. In this sense, lexical frequency constrains accommodation, but its effect depends on whether the task provides a sufficiently direct route from the perceived token to subsequent production.

The third domain consists of speaker-related and social factors. In the present study, accommodation was shaped not only by task and lexical frequency, but also by participant gender and direction of accommodation. Women accommodated more than men overall, but gender effects were not reducible to a single main effect: they also interacted with task, lexical frequency, and direction of change. Direction is important because accommodation from a plosive baseline to a fricative variant and accommodation from a fricative baseline to a plosive variant differ in their sociolinguistic associations. The exploratory questionnaire results further suggest that explicit evaluations of the alternative variant may vary by lexical frequency and direction, although these results remain descriptive and cannot establish a causal role for attitudes. These patterns indicate that speaker-related and social factors may influence accommodation directly, while also affecting how strongly representational factors such as lexical frequency shape accommodation.

The theoretical contribution of these findings is to clarify how the components of a hybrid account may interact. The influence of a recently perceived form on production depends jointly on the directness of the input–output mapping, the representational strength of the forms involved, and speaker-related and sociolinguistic factors. The lexical-frequency result illustrates this interdependence: greater accommodation for low-frequency items emerged under the close input–output coupling of shadowing, and the size of this task-dependent contrast varied with participant gender. Accommodation is therefore best understood not as the product of a single automatic or socially mediated mechanism, but as the outcome of interactions amongst perceived input, stored linguistic representations, processing demands, and characteristics of the speaker and accommodation setting.

### 5.6 Limitations and future directions

Several limitations of the study need to be acknowledged. First, the participant sample is skewed towards female speakers, and the distribution of baseline variants is uneven. As shown in Table 1, both the male subset and the fricative-to-plosive subset are relatively small. This limits the extent to which the present study can draw strong conclusions about effects involving participant gender, especially interactions between gender, lexical frequency, task, and direction of accommodation. The frequency:direction interaction was tested but was not supported; however, given the uneven distribution of baseline variants, the absence of this interaction should not be interpreted as strong evidence that frequency effects are necessarily identical across accommodation directions. Future research should therefore seek a more balanced distribution of participant gender and baseline variants, so that accommodation towards fricative and plosive realisations can be compared more directly.

A related question concerns speakers without a clear dominant baseline variant. Four participants in the present study produced the plosive and fricative variants in approximately equal proportions at baseline and therefore had to be excluded from the accommodation analysis. It would be valuable for future work to examine such speakers directly, since they may provide a particularly informative test case for how speakers accommodate to both variants when neither variant is strongly dominant in their own production.

Second, the interaction task, while more complex than shadowing, remains semi-structured and involves pre-recorded interlocutors. It therefore does not provide a fully naturalistic test of live social interaction. More naturalistic conversational settings may reveal more nuanced patterns of accommodation, including effects of interlocutor identity, social evaluation, and real-time interpersonal dynamics that could not be tested directly in this study.

Third, the present design does not allow the separate components of input–output coupling to be disentangled. As discussed in Sections 2.2 and 5.5, input–output coupling involves at least three partly separable dimensions: the temporal proximity between the perceived input and the produced output, the degree of form similarity or overlap between input and output, and the amount of intervening processing required before production. In the present study, these dimensions covary with task type. Shadowing involved immediate repetition of the same lexical item, with minimal intervening processing. The interaction task, by contrast, placed additional material and processing between the perception of the relevant variant and the production of the target word: participants heard an answer containing one *-ig* adjective, heard a subsequent question, selected an appropriate response, and then produced a different *-ig* adjective. The task difference therefore combined temporal immediacy, processing demands, lexical identity, and overall accommodation strength. Future work should manipulate these components more independently. Temporal proximity could be varied by comparing immediate and delayed repetition while holding lexical identity constant. Form similarity could be manipulated by comparing accommodation to the same word, to a different word with the same suffix, or to an unrelated word containing the same segmental sequence. Intervening processing could be isolated by comparing direct repetition with conditions in which production of the same target word follows a semantic judgement, a question-answering step, or turn planning. Such designs would make it possible to determine whether frequency effects in accommodation depend primarily on the recency of the input, on the degree and type of form similarity, on the amount of intervening processing, or on the overall degree of accommodation elicited by the task.

Fourth, the use of categorical frequency levels simplifies what is in reality a gradient phenomenon. Future studies could employ continuous measures of lexical frequency to capture more fine-grained effects. A further question for future research is whether lexical frequency and contextual predictability make separable contributions to accommodation. Whereas lexical frequency reflects speakers' accumulated experience with a word across contexts, contextual predictability concerns the degree to which that word is expected in the immediate linguistic or discourse environment. Future studies could manipulate these dimensions independently in richer discourse contexts, making it possible to determine whether accommodation is shaped primarily by the representational strength of a lexical item, by local expectations about its occurrence, or by an interaction between the two.

Finally, the analysis is based on a single linguistic variable, the German adjectival suffix *-ig*. This variable is well suited to the present research question because it provides a clear alternation between fricative and plosive realisations, but its sociophonetic status is complex and regionally variable. Further work is therefore needed to establish whether similar task-dependent frequency effects emerge for other variables and other types of phonetic contrast.

## 6 Conclusion

This study demonstrates that phonetic accommodation is systematically shaped by processing conditions rather than occurring as a uniform or context-independent response to perceived speech. Lexical frequency affected accommodation conditionally: low-frequency items elicited greater accommodation than high-frequency items in shadowing, but no comparable frequency effect emerged in interaction. This pattern indicates that frequency effects are strongest when input–output coupling is sufficiently direct to allow a recently perceived pronunciation variant to influence production, especially when perceived and produced forms are lexically identical.

More broadly, accommodation emerged from the interaction of multiple factors. It was stronger under conditions of relatively direct input–output mapping, increased with cumulative exposure, and varied with participant gender and direction of accommodation, with women generally showing greater accommodation than men. By contrast, stimulus voice gender did not play a detectable role in the present design.

The task contrast further suggests that frequency-related effects are most likely to emerge when perceived and produced forms overlap at the word level. When accommodation requires generalising across different lexical items via a shared suffix, as in the interaction task, these effects may be attenuated or fail to emerge. By linking lexical-frequency effects to the relation between perceived and produced forms, task-specific processing demands, the representational strength of linguistic forms, and speaker-related factors, this study contributes to a more integrated account of adaptive speech behaviour.

## Data Availability

The raw data supporting the conclusions of this article will be made available by the authors, without undue reservation.

## Footnotes

1 Grundschule; Mittelstufe (Real-, Gemeinschaftsschule, etc.); Oberstufe.

2 Target adjectives. High frequency: *gültig* ‘valid', *günstig* ‘cheap', *heftig* ‘violent', *kräftig* ‘strong', *künftig* ‘future', *lustig* ‘funny', *nötig* ‘necessary', *richtig* ‘correct', *tätig* ‘employed', *wichtig* ‘important'. Low frequency: *biestig* ‘beastly', *borstig* ‘bristly', *haltig* ‘sustainable', *häutig* ‘skin-like', *krautig* ‘herbaceous', *mistig* ‘crappy', *sichtig* ‘visible', *spritig* ‘fuel-like', *zotig* ‘naughty'.
